# Association between Cardiovascular Response and Inflammatory Cytokines in Non-Small Cell Lung Cancer Patients

**DOI:** 10.3390/jcdd10040173

**Published:** 2023-04-17

**Authors:** Xiaolin Wang, Mengying Cao, Zilong Liu, Liming Chen, Yufei Zhou, Pan Gao, Yunzeng Zou

**Affiliations:** 1 Shanghai Institute of Cardiovascular Diseases, Zhongshan Hospital, Fudan University, Shanghai 200032, China; xiaolinwang20@fudan.edu.cn (X.W.); mycao19@fudan.edu.cn (M.C.); 21111210017@m.fudan.edu.cn (L.C.); zyf15851852592@163.com (Y.Z.); 2 Institute of Biomedical Sciences, Fudan University, Shanghai 200032, China; 3 Department of Pulmonary and Critical Care Medicine, Zhongshan Hospital, Fudan University, Shanghai 200032, China; liu.zilong@zs-hospital.sh.cn; 4 Shanghai Key Laboratory of Bioactive Small Molecules, Fudan University, Shanghai 200032, China

**Keywords:** NSCLC, cardiovascular disease, inflammatory cytokine, biomarker

## Abstract

Cardiovascular disease is an essential comorbidity in patients with non-small cell lung cancer (NSCLC) and represents an independent risk factor for increased mortality. Therefore, careful monitoring of cardiovascular disease is crucial in the healthcare of NSCLC patients. Inflammatory factors have previously been associated with myocardial damage in NSCLC patients, but it remains unclear whether serum inflammatory factors can be utilized to assess the cardiovascular health status in NSCLC patients. A total of 118 NSCLC patients were enrolled in this cross-sectional study, and their baseline data were collected through a hospital electronic medical record system. Enzyme-linked immunosorbent assay (ELISA) was used to measure the serum levels of leukemia inhibitory factor (LIF), interleukin (IL)-18, IL-1β, transforming growth factor-β1 (TGF-β1), and connective tissue growth factor (CTGF). Statistical analysis was performed using the SPSS software. Multivariate and ordinal logistic regression models were constructed. The data revealed an increased serum level of LIF in the group using tyrosine kinase inhibitor (TKI)-targeted drugs compared to non-users (*p* < 0.001). Furthermore, serum TGF-β1 (area under the curve, AUC: 0.616) and cardiac troponin T (cTnT) (AUC: 0.720) levels were clinically evaluated and found to be correlated with pre-clinical cardiovascular injury in NSCLC patients. Notably, the serum levels of cTnT and TGF-β1 were found to indicate the extent of pre-clinical cardiovascular injury in NSCLC patients. In conclusion, the results suggest that serum LIF, as well as TGFβ1 together with cTnT, are potential serum biomarkers for the assessment of cardiovascular status in NSCLC patients. These findings offer novel insights into the assessment of cardiovascular health and underscore the importance of monitoring cardiovascular health in the management of NSCLC patients.

## 1. Introduction

Cardiovascular disease is a significant public health issue worldwide [1]. Antineoplastic therapy-associated cardiotoxicity has attracted widespread attention in recent years [2]. Cancer patients with exiting cardiovascular disease present mortality rates ten times higher than those without [3]. Therefore, protecting cancer survivors from cardiovascular injuries is of great significance. In the United States, the number of cancer patients is expected to increase to 26 million by 2040 [4], making cardiac surveillance of oncology patients crucial. In China, sudden cardiac death related to cancer therapy has been increasing rapidly [5], highlighting the need for preventative measures. Antineoplastic drugs, the unhealthy lifestyles of oncology patients, and the effects of radiotherapy, may lead to cardiovascular injury [6]. The primary manifestation of cardiotoxicity associated with cancer therapy is clinical cardiac dysfunction, as defined by the European Society for Medical Oncology Guidelines Committee, which includes a decrease in left ventricular ejection fraction (LVEF) ≥5–10 to <50–55%, with or without symptoms [7,8].

In addition to echocardiology, serum biomarkers provide a rapid and cost-effective approach for identifying cardiovascular risk in oncology patients [9]. These biomarkers improve the efficiency of risk prediction and classification of oncology patients with cardiotoxicity [10]. Cardiac troponin, a component of the sarcomere—the contractile unit of the heart—isotopes I (cTnI) and T (cTnT) are thought to be cardiac-specific [11]. Clinical studies have shown serum cTnT and cTnI levels are associated with cardiotoxicity in cancer chemotherapy patients [12,13]. Amino-terminal proB-type natriuretic peptide (NT-proBNP), a member of the natriuretic peptide family, secreted into the bloodstream due to myocardial stretching and stress caused by ventricular pressure or volume loading [14], aids in the diagnosis and prediction of heart failure events [15]. Elevated serum NT-proBNP levels during chemotherapy are associated with left ventricular dysfunction and heart failure [16,17,18]. An increasing number of basic and clinical studies have recently shown that inflammatory factors are also involved in the development of cardiotoxicity in oncology patients [19]. Based on pathophysiological development of cardiotoxicity, this study selected biomarkers related to inflammation, cell metabolism, and cell proliferation and differentiation. 

Leukemia inhibitory factor (LIF) is a member of the interleukin 6 family of cytokines, which has multiple ligands and is involved in various signaling pathway processes. In the cardiovascular aspect, LIF plays a protective role in congestive heart failure by activating STAT3 and increasing the survival of hypoxic cardiomyocytes [20,21,22]. LIF also plays a pro-oncogenic role in tumor progression, accompanied by poor prognosis [23,24,25]. However, the association between LIF and cardiotoxicity has not yet been investigated. Interleukin (IL)-18 is a pro-inflammatory cytokine which regulates the T helper response [26]. Increased circulating IL-18 levels have been detected in doxorubicin-induced cardiotoxicity, and an increased serum level of IL-18 has been related to NLRP3-mediated pyroptosis [27,28]. IL-1β is another pro-inflammatory cytokine that plays a significant role in the immune response [29], and an elevated serum level of IL-1β has been associated with a higher risk of cardiotoxicity after doxorubicin treatment [30,31]. Lactate dehydrogenase (LDH) is an enzyme involved in converting glucose to energy [32] and has already been utilized as a biomarker for the clinical diagnosis of cardiotoxicity [33], presenting a dose-independent increase after doxorubicin treatment [34]. Increased serum LDH levels in oncology patients are associated with poor prognosis through glucose accumulation [35]. Secreted frizzled-related protein 2 (Sfrp2) is another marker of cellular metabolism [36], which was initially described as a Wnt antagonist [37]. Sfrp2 has been shown to be decreased in several tumor types as a tumor suppressor, but has also been shown to activate tumor angiogenesis, as a tumor promoter [38]. Recent studies have indicated that Sfrp2 promotes cardiac function and attenuates doxorubicin-induced cardiotoxicity by regulating the Akt/mTOR pathway [39]. Connective tissue growth factor (CTGF) and transforming growth factor β1 (TGFβ1), cytokines regulating cell proliferation, were found to be decreased in doxorubicin-induced cardiotoxicity and were utilized to evaluate the absence of cardiovascular injury in NSCLC patients in our study [40,41].

This study aims to explore the correlation between these serum inflammatory factors and pre-clinical cardiovascular injury in NSCLC patients, in order to determine effective biomarkers for the diagnosis and monitoring of the cardiovascular status in NSCLC patients.

## 2. Materials and Methods

### 2.1. Study Design and Patients

This cross-sectional study recruited NSCLC patients from Zhongshan Hospital, Fudan University (Shanghai, China) from December 2021 to August 2022.

The inclusion criteria were as follows: patients with a confirmed diagnosis of NSCLC stage III or VI; age >16 years and <90 years.

The exclusion criteria were as follows: (1) severe disease progression within the last month; (2) history of coronary heart disease/severe valvular disease/cardiac surgery/vascular surgery/thyroid disease; (3) family history of hypertension, cardiomyopathy or arrhythmia; (4) inability to read or understand the informed consent form. 

The definition of preclinical cardiovascular injury was as follows: patients with one of the following symptoms: (1) simple hypertension; (2) transient ECG abnormalities; (3) cardiac enzyme abnormalities. 

The severity of preclinical cardiovascular injuries means the number of symptoms the patients have: 0, 1, 2, and 3. 

The study was approved by the ethics committees of Zhongshan Hospital, Fudan University (B2020-078R). A total of 118 patients were enrolled in the study to assess the correlation of serum inflammatory biomarkers with cardiovascular performance in NSCLC patients. 

### 2.2. Acquisition of Clinical Information

The patient’s basic information was obtained from the electronic medical record system of Zhongshan Hospital of Fudan University, and the clinical indicators included age, disease stage, medical record type, medication use, cTnT, NTproBNP, cTnT, and other indicators.

### 2.3. Enzyme-Linked Immunosorbent Assay (ELISA)

Blood samples were obtained from individuals in the fasted state. Venous blood was drawn to a serum separator tubed overnight at 4. Blood samples were centrifuged at 3000 rpm for 15 min at room temperature. Then the supernatant was obtained and stored at −80 °C until detection. IL-1β, IL-18, LDH, LIF, CTGF, and TGF-β1 were assessed by ELISA kits (Ybio, Shanghai, China) following the manufacturer’s instructions.

### 2.4. Assessment of Hypertension, ECG Abnormalities and Cardiac Enzyme Abnormalities

The definition of hypertension followed Chinese guidelines for the management of hypertension [42], as follows, the systolic blood pressure ≥140 mmHg or the diastolic blood pressure ≥90 mmHg, without organ lesion.

The definition of ECG abnormalities defined as micro or transient changes that quickly reverted to normal and did not meet the clinical criteria for diagnosing arrhythmias recommended by Recommendations for the Standardization and Interpretation of the Electrocardiogram from the American Heart Association Electrocardiography and Arrhythmias Committee [43]. In our study, ECG abnormalities mainly includes ST segment or T segment changes.

### 2.5. Statistical Analyses

Multivariate logistic regression models were used to test the association of sex, age (per SD increase), cTnT (per SD increase), NT-proBNP (per SD increase), TGF-β1 (per SD increase), and other indicators with preclinical cardiovascular injury. Similarly, ordinal logistic regression models were used to test the association of indexes (increased per SD) with severity of preclinical cardiovascular injury.

ROC curve was plotted using the SPSS software and its AUC value was obtained. The cut-off value was determined based on the calculated Youden’s index [44,45]. 

Statistical analysis was performed using SPSS software (SPSS Statistics for Windows, version 20.0). (SPSS Inc.: Chicago, IL, USA). Continuous variables were expressed as means and standard deviations. Associations between continuous variables were tested by Pearson’s correlation coefficient. The strength levels of correlation were weak (0–0.25), moderate (>0.25–0.50), strong (>0.50–0.75), and very strong (>0.75) [46]. Differences between the two groups were assessed using the Student’s t-test (normally distributed data) or the Mann-Whitney test (non-normally distributed). *p*-value < 0.05 was considered to be statistically significant. 

## 3. Results

### 3.1. Baseline Patient Characteristics

Patients with NSCLC (*n* = 118) were recruited for this study. The baseline characteristics of the patients are presented in Table 1. To observe the cardiovascular response to TKI-targeted drugs, the patients were divided into two groups, one that received TKI-targeted therapy (*n* = 40) and the other that did not receive TKI-targeted therapy (*n* = 78) (Table 1). The mean age of patients who received TKI-targeted therapy was 58.0 ± 13.5 years, while the mean age of patients who did not receive TKI-targeted therapy was 61.5 ± 8.4 years. Five patients with a history of smoking were observed in the group that did not receive TKI-targeted therapy, while none of the patients in the TKI-targeted therapy group had a history of smoking (*p* = 0.165). There was no statistically significant difference between the two groups in terms of disease staging and clinical condition. However, there was a statistically significant difference in the gender distribution between the group that did not receive TKI-targeted therapy (63 males and 15 females) and the targeted therapy group (24 males and 16 females) (*p* = 0.0001). The mean serum concentration of TIBL in the TKI-targeted group was 7.7 μmol/L, in contrast to 6.6 μmol/L in the control group (*p* < 0.05). The carcinoembryonic antigen (CEA) of patients ranged from 2.3 to 137 ng/mL, indicating varying severity in the included patients. There was no statistically significant difference in the CEA levels between the two groups. Regarding serum biomarkers, a significant decrease in LIF level was observed in patients who received TKI-targeted therapy (*p* < 0.01), while no significant differences were observed in the remaining serum biomarkers including TGF-β1, IL-6, and tumor necrosis factor-alpha (TNF-α).

### 3.2. Correlation between Serum LIF Levels and Clinical Features

**Figure 1:**
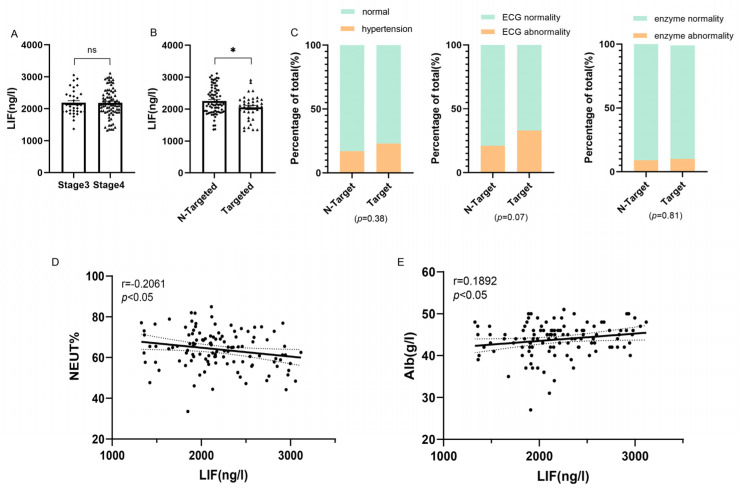
Correlation between serum leukemia inhibitory factor (LIF) levels and clinical features. (**A**): Serum LIF levels show no significance between different non-small cell lung cancer (NSCLC) stages. (**B**): Serum LIF levels decreased in the tyrosine kinase inhibitors (TKI)-targeted group. (**C**): The different proportions of hypertension, and electrocardiogram (ECG) abnormality between the two groups. (N-Targeted = 78, Targeted = 40) (**D**,**E**): Correlation analysis between serum LIF levels and clinical features. (*n* = 118) (neutrophil count (NEUT), albumin (Alb),* *p* < 0.05, ns, no significance).

First, no significant difference in serum LIF expression level between stage III and stage IV NSCLC was found (Figure 1A). Serum LIF levels were remarkably lower in the TKI-targeted therapy group than in the non-targeted therapy group (Figure 1B). As shown in Figure 1C, the proportion of hypertension and ECG abnormalities were slightly higher in the TKI-targeted therapy group than in the non-targeted therapy group in this study, while the proportion of myocardial enzyme abnormalities was approximately the same in the two groups. In addition, the correlation between LIF and some clinical indicators was analyzed and LIF was associated with the extent of disease deterioration and health status. Serum LIF levels were negatively correlated with the neutrophil ratio (r = −0.2061, *p* < 0.05; Figure 1D). Additionally, serum LIF levels were positively related with serum albumin (r = 0.1982, *p* < 0.05; Figure 1E).

### 3.3. Correlation between Serum LIF Levels and Inflammatory Biomarkers

**Figure 2:**
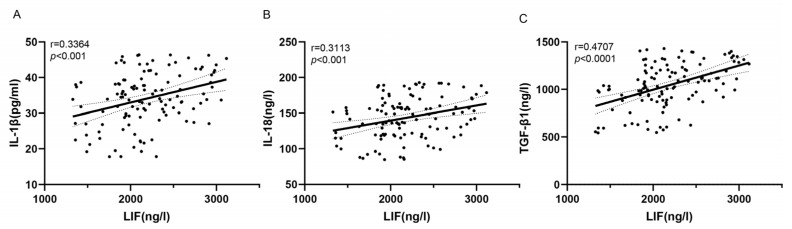
(**A**–**C**): Correlation analysis between serum LIF and other serum cytokines. (*n* = 118) (interleukin (IL), transforming growth factor-β1 (TGF-β1)).

We next explored the association between LIF and other inflammatory cytokines. As shown in Figure 2, LIF was positively correlated with IL-1β (r = 0.3364, *p* < 0.001; Figure 2A) and IL-18 (r = 0.3113, *p* < 0.0001; Figure 2B) and TGF-β1 (r = 0.4707, *p* < 0.0001; Figure 2C).

### 3.4. Multivariable Logistic Regression Models

Univariate and multivariate logistic regression models were constructed to identify effective factors suggesting pre-clinical cardiovascular injury. Age, gender, tumor stage, pathological type, and other indicators were included in the multivariate logistic regression model. The variables of cTnT and TGF-β1 were found to be statistically significant in predicting pre-clinical cardiovascular injuries. In the adjusted model, cTnT was found to be positively associated with pre-clinical cardiovascular injury in NSCLC patients (OR: 1.791 × 10^88^; 95% CI: 6.706 × 10^27^–4.785 × 10^148^), while TGF-β1 (OR: 1.004; 95% CI: 1.0011–1.008) was slightly positively related to pre-clinical cardiovascular injury. The variables of LIF, hypersensitive C-reactive protein (hsCRP), IL-1β, and CTGF were not found to be associated with pre-clinical cardiovascular status, as shown in Table 2.

### 3.5. Ordinal Logistic Regression Models

In the adjusted model, TGFβ1 was found to be positively associated with the extent of pre-clinical cardiovascular injury in NSCLC patients (OR: 0.004; 95% CI: 00.001–0.007). Furthermore, cTnT (OR: 156.714; 95% CI: 65.190–248.237) was strongly correlated with the severity of pre-clinical cardiovascular injury. However, LIF, hsCRP, IL-1β, and other clinical and serum indices were not observed to be associated with pre-clinical cardiovascular status (Table 3). 

### 3.6. The Clinical Value of Serum TGF-β1 Levels for Indicating Preclinical Cardiovascular Performance in NSCLC Patients

**Figure 3:**
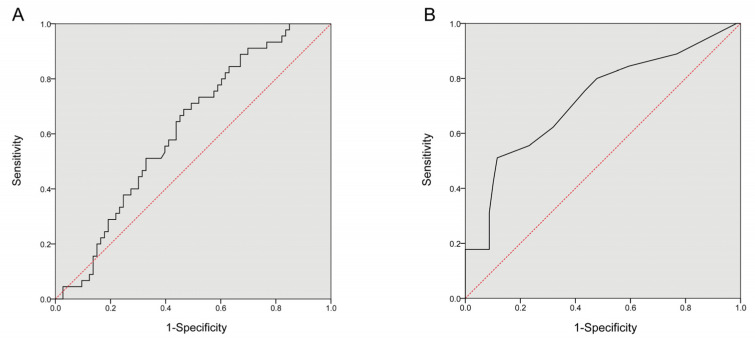
The clinical value of TGF-β1 and cardiac troponin T (cTnT)**.** (**A**)**:** Receiver operating characteristic (ROC) curve about the ability of serum TGF-β1 levels evaluating preclinical cardiovascular injury in NSCLC patients (*p* = 0.035, the Youden’s index = 0.223). (**B**): ROC curve about the clinical value of cTnT for preclinical cardiovascular injuries in NSCLC patients (*p* < 0.0001, the Youden’s index = 0.395).

Next, the diagnostic value of serum TGF-β1 levels for identifying pre-clinical cardiovascular injury in NSCLC patients was analyzed using receiver operating characteristic (ROC) curve analysis. The sensitivity and specificity of TGF-β1 were 68.9% and 53.4% (cutoff point, 1016.158 pg/mL; area under the curve (AUC) value, 0.616; see Figure 3A). 

### 3.7. The Clinical Value of Serum cTnT Levels for the Performance of Assessing Preclinical Cardiovascular Injuries in NSCLC Patients

The ROC curve analysis indicated that serum cTnT had a sensitivity of 51.1% and a specificity of 88.4%, respectively, in evaluating pre-clinical cardiovascular injury in NSCLC patients (cutoff point, 0.0105 ng/mL; AUC value, 0.720; see Figure 3B).

## 4. Discussion

In this cross-sectional and observational study, we collected data from NSCLC patients to explore the distribution of cardiotoxicity and associated risk factors by describing their clinical data and analyzing the related relationships, aiming to identify potential biomarkers that could signify the immediate cardiovascular condition of NSCLC patients. The current study observed changes in inflammatory cytokines in the TKI-targeted group, which has been associated with cardiovascular injury. Cardiovascular disease is one of the leading causes of mortality among long-term cancer survivors [3]. The risk of cardiovascular injury in NSCLC patients depends on various factors such as drug therapy and radiation history. Recent studies have shown that TKI-targeted therapy was accompanied by cardiotoxicity, which manifest as hypertension, arrhythmias, and heart failure [47,48]. In this study, there was a slight increase in the proportion of hypertension and ECG abnormalities in the TKI-targeted therapy group. However, the scale of this research needs to be further expanded to reach a more precise conclusion. Cardiotoxicity related to TKI-targeted therapy includes both direct and indirect aspects [48]. In direct targeted toxicity, the major kinase targets of TKI, such as adenosine triphosphate and vascular endothelial growth factor receptor, are involved in signaling pathways that are important for cardiomyocyte growth and survival [49,50]. However, many TKIs non-specifically inhibit other kinases besides the primary target. For instance, ponatinib primarily exerts its cardiotoxicity through an off-target effect on cardiomyocyte pro-survival signaling pathways by negatively inhibiting protein kinase B and extracellular regulated protein kinases [17,51]. The complexity of tyrosine kinase signaling pathways and the off-target effects of multiple other kinases targeted by these drugs make it challenging to identify the specific molecular mechanisms underlying their cardiotoxicity [52]. Further studies are required to elucidate the mechanisms of TKI-induced cardiotoxicity.

In this study, we found for the first time that TKI-targeted drug treatment caused a decrease in serum LIF levels. This reduction in LIF suggested a potential cancer-suppressive effect of TKI treatment, as well as a potential for cardiovascular damage [53]. LIF is a member of the interleukin 6 family of cytokines. The LIF receptor complex, composed of LIF, LIFR, and glycoprotein 130 (gp130), is responsible for promoting tumor growth, progression, and metastasis through the direct effect of JAK/STAT3 and other downstream pathways, such as MAPK, AKT, and mTOR [54]. This receptor complex is also involved in the development of tumor malignancy, which is a sign of poor prognosis [55]. In terms of cardiovascular aspects, LIF treatment could reduce myoblast cell apoptosis and increase the transcriptional levels of Bcl-xL and XIAP [56]. Furthermore, a sharp decrease in serum LIF was noticed in mice after myocardial infarction (MI) [57]. However, the effect and mechanism of LIF in cardiotoxicity in cancer progression and treatment remain unclear. In the present study, serum LIF levels were significantly reduced in the TKI-targeted treatment group of NSCLC patients, possibly due to the participation of LIFR in the binding of gp130 to the JAK-Tyk family of cytoplasmic tyrosine kinases [58], the detailed mechanism of which should be further explored. Through further correlation analysis, we found that LIF was positively correlated with serum indicators (IL-18, IL-1β, and TGF-β1) and albumin, and negatively correlated with the neutrophil ratio. There was no significant difference in LIF level in the binary analysis of hypertension, ECG abnormalities, and tumor stage, which may need to be further confirmed by expanding the sample size. Overall, LIF plays an important pathophysiological role in cancer and cardiovascular diseases, and may be considered as a new inflammatory biomarker to monitor the status of tumor progression and underlying cardiovascular damage in NSCLC patients.

Our findings revealed at least two potential serum biomarkers indicating pre-clinical cardiovascular injury in NSCLC patients. These findings, along with previous studies linking cardiovascular injuries to NSCLC patients, emphasize the importance of targeted surveillance of individuals with NSCLC to improve early detection of cardiovascular risk. To identify potential serum biomarkers for pre-clinical cardiovascular injury in NSCLC patients, we used multivariate logistic regression and found that serum TGF-β1 levels could serve as a simple and effective indicator. The ordinal logistic regression model also demonstrated TGFβ1 was positively related to the severity of pre-clinical cardiovascular injury. TGF-β1 is a multi-functional cytokine which plays a role in regulating cell proliferation, differentiation, adhesion, migration, and immune monitoring [59,60,61,62]. TGF-β1 was initially thought to be effective in inhibiting cell growth by regulating cyclin-dependent kinases (CDKs) [63]. However, several independent studies have demonstrated that this cytokine is highly expressed in many tumor tissues and is correlated with late-stage cancer and decreased patient survival [64,65,66]. This observation is attributed to the resistance of tumor cells to its negative regulatory effect during the pathological process or in the tumor microenvironment [66,67]. In our study, we found that TGF-β1 could be a potential serum biomarker for assessing pre-clinical cardiovascular injury in NSCLC patients, possibly for the following reasons: on the one hand, TGF-β1 activates the expression of collagen I and IL-11 through RAS/MAPK and PI3K regulation, as well as promoting the fibrotic process [68,69,70]; on the other hand, TGF-β1 may aggravate cardiovascular dysfunction by reducing endoplasmic reticulum ROS production, inhibiting antioxidant enzyme systems, and promoting ventricular remodeling [60,71], thereby reducing patients’ survival [72]. To validate TGF-β1 as a potential serum biomarker for pre-clinical cardiovascular injury monitoring in NSCLC patients, we determined the cutoff point of serum TGF-β1 levels and validated TGF-β1 using ROC curves for NSCLC patients (the cutoff point was 1016.158 pg/mL, while the AUC value was 0.616). Therefore, the serum TGF-β1 levels could serve as a potential serum biomarker for pre-clinical cardiovascular injury monitoring in NSCLC patients.

Our results showed that cTnT levels were positively correlated with the appearance and extent of pre-clinical cardiovascular injury in NSCLC patients. Therefore, we evaluated the clinical value of cTnT indicating pre-clinical cardiovascular injury in NSCLC patients through ROC curve (the cutoff point was 0.0105 ng/m, while the AUC value was 0.720). The results suggest that monitoring serum cTnT levels may be useful in assessing the cardiovascular status of NSCLC patients. Our study is consistent with previous research demonstrating that cancer survivors are more likely to have elevated hs-cTnT levels than healthy individuals. Elevated serum hs-TnT levels have been associated with high mortality rates in oncology patients [73,74,75]. Different from the typical diagnosis of cardiotoxicity in cancer survivors, the definition of pre-clinical cardiovascular injury encompasses several aspects, as explained above. Our findings suggest that increased serum cTnT levels indicate poor pre-clinical cardiovascular health in NSCLC patients. Moreover, our ordinal logistic regression data revealed that patients with high cTnT levels demonstrated greater pre-clinical cardiovascular severity. In conclusion, our study provides further evidence of the clinical value of serum cTnT levels as a biomarker for pre-clinical cardiovascular performance in NSCLC patients.

This study provides a possible approach for timely monitoring the occurrence of pre-cardiovascular injury in NSCLC patients and lays the foundation for subsequent research on tumor-induced cardiotoxicity. The current study has some limitations. One major limitation was the lack of cardiac functional data since echocardiography is not a routine examination for cancer patients. As a result, the study was unable to investigate the impact of these biomarkers on cardiac function. However, we did collect data on various clinical parameters, such as cTNT and NT-proBNP, which may provide insight into the potential effects of these biomarkers on cardiac function. Another limitation was that the cross-sectional study prevented the researchers from making causal conclusions. In our future research, we will consider incorporating other research designs, such as experimental or quasi-experimental designs, to further strengthen our findings. Additionally, we will continue to explore ways to increase our sample size to enhance the generalizability of our results. In addition, the study lacked healthy controls, which could potentially impact the significance of the serum biomarkers in identifying cardiovascular conditions in NSCLC patients. Inflammation levels are generally higher in NSCLC patients than in healthy individuals, and this could have influenced our results. Further research is necessary to confirm the role of LIF, TGF-β1, and cTnT biomarkers in determining cardiovascular health in NSCLC patients. 

## 5. Conclusions

In conclusion, this study highlights the potential of serum biomarkers as a valuable tool for monitoring cardiovascular injury in NSCLC patients. Specifically, the findings demonstrate that serum LIF levels are associated with TKI-targeted therapy, while serum TGF-β1 and cTnT levels are related to preclinical cardiovascular injuries in NSCLC patients. The identification of these associations provides important insights into the potential use of serum biomarkers for monitoring cardiovascular health in NSCLC patients. Overall, this study adds to the growing body of evidence supporting the use of serum biomarkers in the monitoring and management of cardiovascular disease in NSCLC patients.

## Figures and Tables

**Table 1:** Characteristics of patients with NSCLC in different treatments.

	N-Targeted (*n* = 78)	Targeted (*n* = 40)	*p*-Value
Age (years)	61.5 ± 8.4	58.0 ± 13.5	0.124
Gender (M/F)	63/15	24/16	0.0001
Smoking history	5	0	0.165
Stage (IIIA, IIB, IIIC, IVA, IVB)	15, 10, 7, 25, 21	3, 1, 1, 11, 24,	0.005
Histology type (Adenocarcinoma/squamous cell carcinoma/others)	39, 32, 7	36, 0, 4	<0.0001
Clinical condition (PR, SD, PD)	35, 40, 3	12, 25, 3	0.251
Sequential treatment	4	2	0.672
HGB (×10^9^ g/L)	128.0 ± 17.2	130.2 ± 15.3	0.498
WBC (×10^9^ g/L)	5.6 (4.5–6.9)	5.6 (4.9–7.6)	0.472
PLT (g/L)	212.0 (163.0–263.3)	199.0 (164.8–282.8)	0.886
NEUT (%)	63.1 ± 9.7	65.6 ± 8.2	0.177
Albumin (g/L)	44.0 ± 3.3	43.5 ± 5.1	0.597
NSE (ng/mL)	14.5 (12.2–16.7)	15.2 (12.2–18.5)	0.688
TBIL (umol/L)	6.6 (5.4–8.6)	7.7 (6.6–11.5)	0.027
Cre (umol/L)	79.0 (70.0–93.0)	85.0 (69.8–103.3)	0.462
CEA (ng/mL)	3.85 (2.5–8.25)	13.7 (2.3–137.0)	0.050
CA-199 (U/mL)	10.6 (7.7–24.8)	19.3 (10.1–31.2)	0.088
CK19 (ng/mL)	2.6 (1.6–4.7)	3.2 (2.0–5.8)	0.169
SCCA (ng/mL)	1.4 (0.9–2.1)	1.3 (0.9–2.1)	0.854
hsCRP (mg/L)	4.0 (1.3–7.8)	3.5 (1.1–13.3)	0.952
cTnT (ng/mL)	0.008 (0.005–0.011)	0.008 (0.005–0.011)	0.566
NTproBNP (pg/mL)	43.2 (19.3–103.5)	49.1 (20.8–73.1)	0.584
IL-1β (pg/mL)	34.6 ± 7.8	32.9 ± 6.8	0.259
IL-18 (ng/L)	142.3 ± 9.5	145.4 ± 29.2	0.587
LDH (ng/L)	42.3 ± 6.3	41.4 ± 6.1	0.503
LIF (ng/L)	2255.0 ± 437.4	2032.3 ± 389.4	0.008
CTGF (ng/L)	2818.2 ± 430.5	2858.8 ± 471.6	0.640
Sfrp2 (ng/L)	170.3 ± 44.4	173.4 ± 37.6	0.804
TGF-β1 (ng/L)	1038.9 ± 231.9	1047.3 ± 243.2	0.854

**Table 2:** Multivariable logistic regression results about the association between inflammatory biomarkers and preclinical cardiovascular injury in NSCLC patients.

	OR	*p*-Value	95% CI	
			Lower limit	Upper limit
Male gender	0.710	0.621	0.183	2.763
TKI-targeted	1.297	0.693	0.356	4.723
Smoking history	0.879	0.927	0.056	13.733
cTnT	1.791 × 10^88^	0.004	6.706 × 10^27^	4.785 × 10^148^
hsCRP	0.994	0.769	0.953	1.036
IL-1β	0.989	0.807	0.904	1.082
IL-18	0.989	0.299	0.969	1.010
LDH	0.968	0.502	0.881	1.064
Sfrp2	1.003	0.694	0.988	1.019
LIF	1.000	0.548	0.998	1.001
TGF-β1	1.004	0.011	1.001	1.008
CEA	0.999	0.735	0.992	1.006
CA199	1.000	0.884	0.999	1.001
NSE	0.965	0.461	0.879	1.060
Cyfra 21-1	1.133	0.133	0.963	1.333
SCCA	0.587	0.109	0.306	1.126

**Table 3:** Ordinal logistic regression results about the association between inflammatory biomarkers and preclinical cardiovascular injury in NSCLC patients.

	OR	*p*-Value	95% CI	
			Lower limit	Upper limit
Male gender	−0.143	0.800	−0.125	0.963
N-TKI-targeted	−0.309	0.561	−1.351	0.732
N-Smoking history	0.190	0.884	−2.368	2.749
cTnT	156.714	0.001	65.190	248.237
hsCRP	−0.001	0.951	−0.30	0.028
IL-1β	0.029	0.453	−0.046	0.103
IL-18	−0.022	0.015	−0.040	−0.004
LDH	−0.025	0.530	−0.101	0.052
Sfrp2	−0.002	0.790	−0.015	0.011
LIF	0.000	0.492	−0.002	0.001
TGF-β1	0.004	0.005	0.001	0.007
CEA	0.001	0.686	−0.003	0.004
SCCA	−0.422	0.060	−0.861	0.017
Threshold 0	1.958	0.488	−3.581	7.497
Threshold 1	4.372	0.126	−1.227	9.971
Threshold 2	8.685	0.007	2.337	15.033

## Data Availability

The datasets used and analyzed during the current study are available from the corresponding author on reasonable request.
